# Melatonin ameliorates the advanced maternal age-associated meiotic defects in oocytes through the SIRT2-dependent H4K16 deacetylation pathway

**DOI:** 10.18632/aging.102703

**Published:** 2020-01-24

**Authors:** Congyang Li, Xi He, Zhenyue Huang, Longsen Han, Xinghan Wu, Ling Li, Yongan Xin, Juan Ge, Jiahao Sha, Zhiqiang Yin, Qiang Wang

**Affiliations:** 1 State Key Laboratory of Reproductive Medicine, Suzhou Municipal Hospital, Department of Histology and Embryology, Nanjing Medical University, Nanjing, China; 2 Center for Global Health, School of Public Health, Nanjing Medical University, Nanjing, China; 3 Department of Dermatology, The First Affiliated Hospital of Nanjing Medical University, Nanjing, China

**Keywords:** melatonin, aging, oocyte quality, meiosis, histone acetylation

## Abstract

It has been widely reported that advanced maternal age impairs oocyte quality. To date, various molecules have been discovered to be involved in this process. However, prevention of fertility issues associated with maternal age is still a challenge. In the present study, we find that both *in vitro* supplement and *in vivo* administration of melatonin are capable of alleviating the meiotic phenotypes of aged oocytes, specifically the spindle/chromosome disorganization and aneuploidy generation. Furthermore, we identify SIRT2 as a critical effector mediating the effects of melatonin on meiotic structure in old oocytes. Candidate screening shows that SIRT2-controlled deacetylation of histone H4K16 is essential for maintaining the meiotic apparatus in oocytes. Importantly, non-acetylatable-mimetic mutant H4K16R partially rescues the meiotic deficits in oocytes from reproductive aged mice. In contrast, overexpression of acetylation-mimetic mutant H4K16Q abolishes the beneficial effects of melatonin on the meiotic phenotypes in aged oocytes. To sum up, our data uncover that melatonin alleviates advanced maternal aged-associated meiotic defects in oocytes through the SIRT2-depenendet H4K16 deacetylation pathway.

## INTRODUCTION

The reproductive aging process is thought to be dominated by a gradual decrease in both the quantity and the quality of the oocytes residing within the follicles present in the ovarian cortex [1]. The age-related decline in oocyte quality is associated with increased spindle abnormalities and aneuploidy [2]. Meiotic spindle/chromosome defects in oocytes of women of advanced maternal age lead to the elevated rates of infertility, miscarriage, and birth defects. To date, various molecules have been discovered to be involved in this process. However, prevention of fertility issues associated with maternal age is still a challenge.

Melatonin (N-acetyl-5-methoxytryptamine), a natural hormone synthesized by the mammalian pineal gland [3] and many other tissues including bone marrow [4], cumulus-oocyte complex [5, 6], thymus [7], and testis [8]. Melatonin has been shown to exert various biological activities, such as antioxidation [9], immune defense [10], and anticancer effects [11]. The importance of melatonin in aging and age-related diseases is underlined by the decreased production of this potent antioxidant in the pineal gland [12, 13], as well as the reduction in melatonin receptor expression in extrapineal tissues [14]. Recent findings suggest that melatonin influences the autophagy processes due to its role as a metabolic regulator and mitochondrial protector [15]. Of note, in the pancreas of senescence accelerated mouse prone-strain 8 (SAMP8), melatonin increased the levels of aging-related genes including *Pdx1*, *FoxO1*, *FoxO3A*, and *Sirtuins* [16]. Sirtuins are a family of NAD^+^-dependent deacetylases (SIRT1-7) that catalyze post-translational modifications of proteins. They have been reported to respond to metabolic challenges or oxidative stress associated with aging [17]. Recently, SIRT1, SIRT2 and SIRT3 have emerged as protectors of oocyte against maternal aging [17]. In eukaryotes, 147 bp of DNA is wrapped around an octamer of histones consisting of two copies of H2A, H2B, H3 and H4. Previous studies indicate that SIRT2 and its yeast counterpart Hst2 have a strong preference for histone in their deacetylation activity [18]. Histone acetylation is crucial for cell cycle control, DNA repair, gene expression, and chromosome architecture/stability [19–22]. In addition, SIRT2 also has been found to regulate cell differentiation via deacetylating various transcription factors [23].

In the present study, we investigated the potential mechanism mediating the effects of melatonin on oocyte quality using a natural aging mouse model. Our data indicate that melatonin supplement was able to markedly decrease the incidence of meiotic defects in oocytes from old mice. Moreover, we found that this protective effect was mediated by the SIRT2-dependent histone H4K16 deacetylation pathway.

## RESULTS

### Melatonin administration alleviates the meiotic defects of oocytes from old mice

It has been widely reported that aged oocytes are more likely to produce abnormal spindle and aneuploidy, which lead to a decreasing chance of fertilization and an increasing risk of miscarriage or of a child with birth defects [24–26]. Melatonin has been proven to possess extensive biological activities including antioxidant, anti-aging, and anti-apoptotic [10, 27]. We postulated that melatonin might provide a beneficial effect against defective phenotypes of aged oocytes.

**Figure 1:**
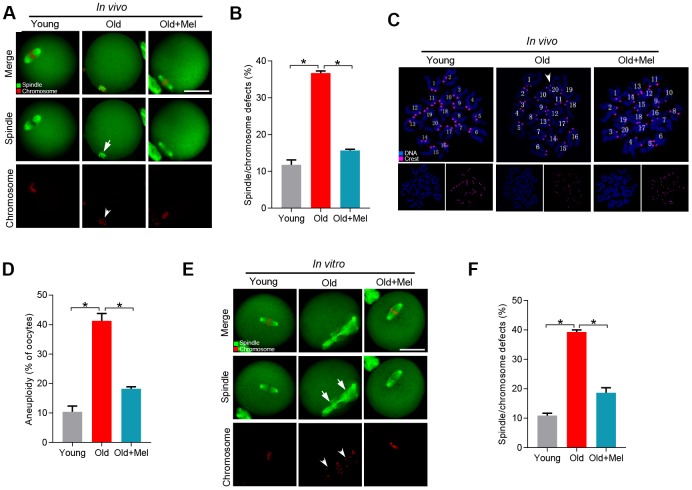
**Effects of melatonin administration on meiotic apparatus in aged oocyte.** (**A**) MII oocytes from young (n=90), old (n=88) and old mice treated with melatonin (old+Mel, n=80) were stained with α-tubulin to visualize spindle (green) and counterstained with propidium iodide to visualize chromosomes (red). Representative confocal sections are shown. Arrows indicate the spindle defects and arrowheads point to the misaligned chromosomes. (**B**) Quantification of young, old and old+Mel oocytes with abnormal spindle and chromosomes. (**C**) Chromosome spreading of young, old and old+Mel MII oocytes with aneuploidy. Chromosomes were stained with Hoechst 33342 (blue), and kinetochores were labeled with CREST (purple). (**D**) Histogram showing the incidence of aneuploidy in young (n=82), old (n=57) and old+Mel (n=53) oocytes. (**E**) Young and old oocytes *in vitro* cultured with or without melatonin were processed to evaluate meiotic apparatus. Young, old, and old+Mel oocytes were stained with α-tubulin to visualize spindle (green) and counterstained with propidium iodide to visualize chromosomes (red). Representative confocal sections are shown. Arrow indicates the disorganized spindle and arrowhead indicates the misaligned chromosomes. (**F**) Quantification of young (n=131), old (n=78) and old+Mel (n=95) oocytes with abnormal spindle/chromosomes. Data are expressed as mean percentage ± SD from three independent experiments. *P<0.05 vs. controls. Scale bar: 50 μm.

To test this hypothesis, female ICR mice (42-45 weeks old) were treated with oral administration of melatonin. Their oocytes were recovered from the oviduct, immunolabeled with α-tubulin antibody to visualize spindle, and costained with propidium iodide for chromosomes. We observed that most oocytes obtained from young mice displayed a typical bipolar spindle as chromosomes congressed to metaphase plate (with only 11.8% abnormal), in contrast around 36.7% of oocytes collected from old mice showed the disorganized spindle and misaligned chromosomes. Interestingly, only 15.7% of meiotic defects were detected in metaphase oocytes from reproductive aged mice administrated with melatonin, which is significantly reduced as compared to those old oocytes (Figure 1A–1B). Meanwhile, we analyzed the karyotype of metaphase II (MII) oocytes by chromosome spreading combined with kinetochore labeling. As shown in Figure 1C–1D, reproductive aged mice showed about 3-fold increase in incidence of aneuploid eggs in comparison with young mice. It is worth noting that the aneuploidy rate of oocytes obtained from reproductive aged mice treated with melatonin decreased significantly. Consistent with this observation, we found that melatonin supplementation during *in vitro* culture also significantly ameliorated the meiotic errors in aged oocytes (Figure 1E–1F). Altogether, these date indicate that melatonin administration could improve the quality of old oocytes, specifically the meiotic phenotypes.

### Melatonin supplementation promotes the protein expression of SIRT2 in oocytes from old mice

**Figure 2:**
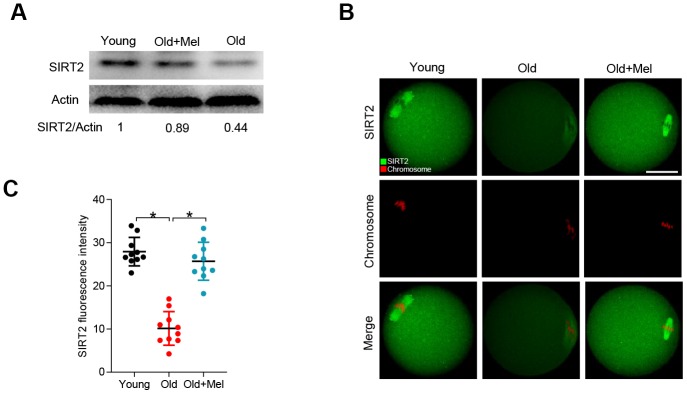
**Effects of melatonin administration on SIRT2 expression in oocytes from old mice.** (**A**) Western blot analysis shows the SIRT2 expression in GV oocytes from young, old and old+Mel mice. Actin served as a loading control. Band intensity was calculated using ImageJ software. (**B**) Representative confocal images of young, old and old+Mel MII oocytes stained with SIRT2 antibody (green) and counterstained with propidium iodide (red) for chromosomes. (**C**) Quantification of the relative fluorescence intensity of SIRT2 in oocytes in (**B**). Each data point represents an oocyte (n=10 for each group). *P<0.05 vs. controls. Scale bar: 50 μm.

Our previous studies have shown that the levels of SIRT2, a regulator of meiotic structure in oocytes, were lowered in oocytes from old mice [28]. Recently, Xu et al. also found that SIRT2 inhibition led to the poor quality of bovine oocytes [29]. Given these findings, we asked whether SIRT2 serves as a potential substrate mediating the effects of melatonin on quality control of aged oocytes. To do this, we examined the SIRT2 expression in young, old and melatonin-treated old (old+Mel) oocytes by performing Western blot. As shown in Figure 2A, the expression of SIRT2 protein in oocytes from reproductive aged mice was reduced by more than 50% compared with young controls, in line with the previous report [28]. Notably, melatonin supplement was able to markedly increase the SIRT2 expression in oocytes from reproductive aged mice. In addition, we observed the similar results by conducting immunofluorescent staining, showing the elevated SIRT2 fluorescence signals in old oocyte when treated with melatonin (Figure 2B–2C). These data together imply that SIRT2 may be the potential target mediating the effects of melatonin on oocyte competence.

### SIRT2 mediates the melatonin effects on meiotic structure in old oocytes

**Figure 3:**
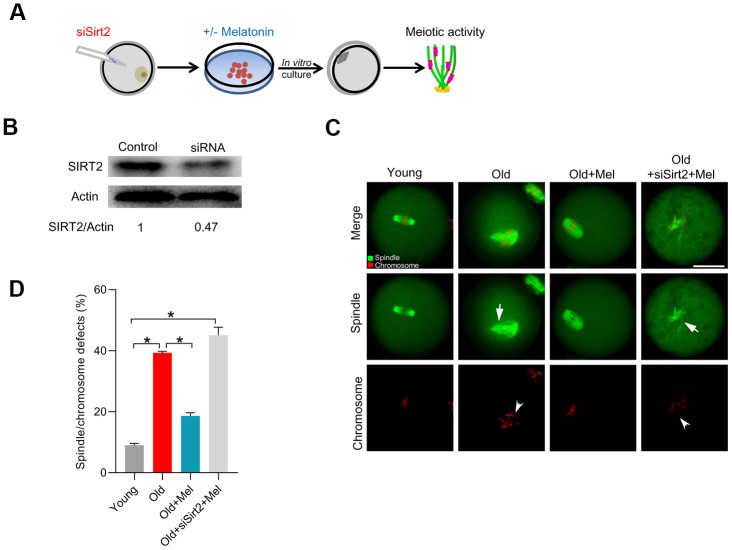
**SIRT2 knockdown abolishes the effects of melatonin on meiotic phenotypes in aged oocytes.** (**A**) Schematic illustration of the experimental protocol to check whether SIRT2 mediates the effects of melatonin on aged oocyte. (**B**) The efficiency of SIRT2 knockdown was verified by Western blot. Actin served as a loading control. Band intensity was calculated using ImageJ software. (**C**) Representative images of spindle/chromosome organization in young, old, old+Mel and old+Mel+siSirt2 oocytes are shown. Arrows indicate the disorganized spindle and arrowheads indicate the misaligned chromosomes. (**D**) Quantification of young, old, old+Mel and old+Mel+siSirt2 oocytes with abnormal spindle/chromosomes. Error bars indicate ± SD. *P<0.05 vs. controls. Scale bars: 50 μm.

To investigate whether the action of melatonin on the quality of aged oocytes was through the regulation of SIRT2 expression, we depleted SIRT2 in old oocytes with specifically-designed siRNA (siSirt2), and then checked whether melatonin still can alleviate the deficient phenotypes of old oocytes, as illustrated in Figure 3A. As shown in Figure 3B, the knockdown efficiency of siRNA was verified by immunoblotting. Next, we evaluated the meiotic phenotypes of matured oocytes, specifically the spindle/chromosome organization. As shown in Figure 3C–3D, melatonin administration was unable to lower the frequency of spindle defects and chromosome misalignment in old oocytes following SIRT2 knockdown. Collectively, these findings indicate that SIRT2 serves as a critical effector mediating the beneficial effects of melatonin on meiotic structure in old oocytes.

### Melatonin supplementation lowers the acetylation level of H4K16 in old oocytes

**Figure 4:**
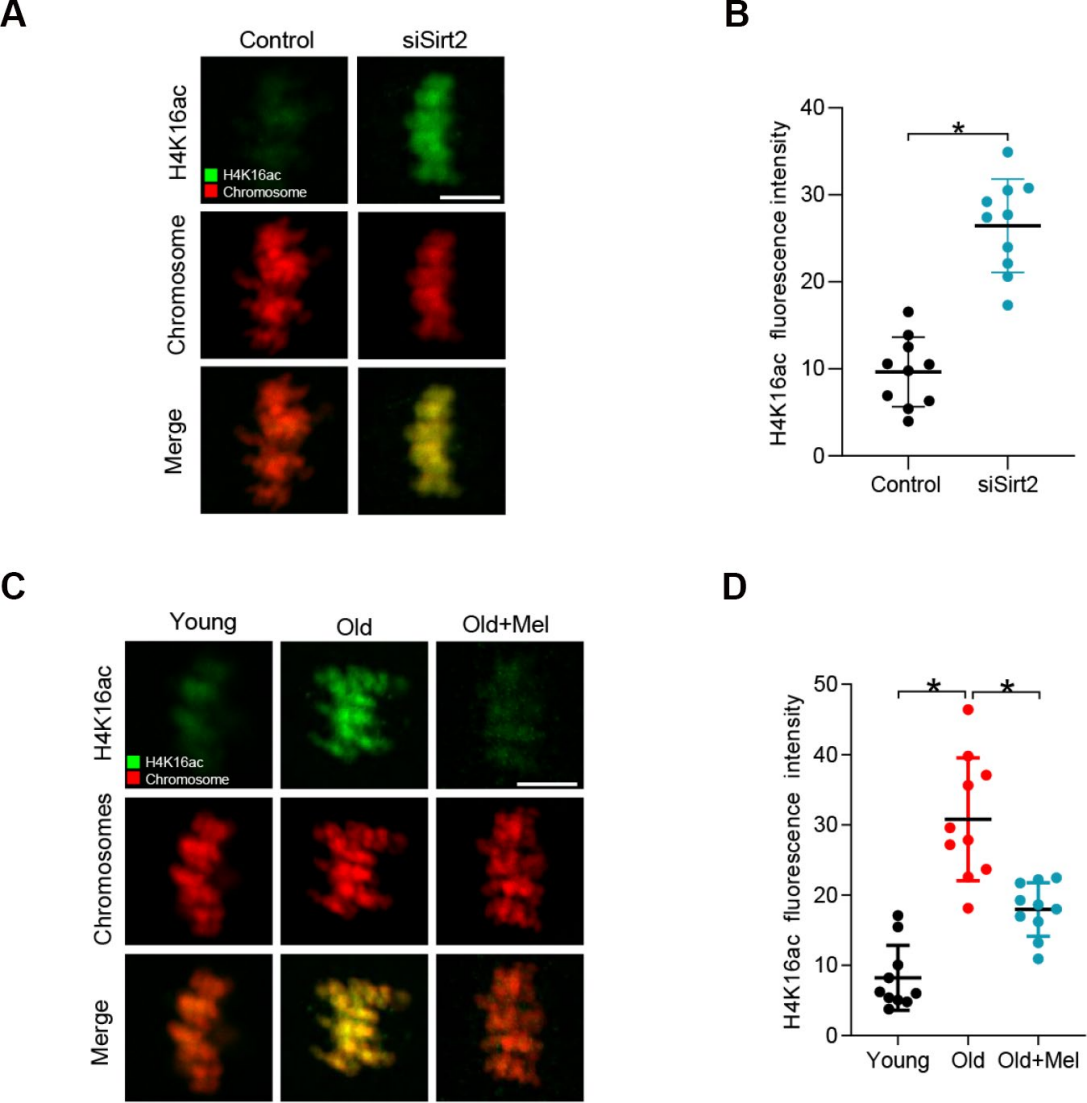
**H4K16 hyperacetylation in old and SIRT2-depleted oocytes.** (**A**) Control and SIRT2 knockdown (siSirt2) oocytes were immunolabeled with H4K16ac antibody (green) and counterstained with propidium iodide (red) for DNA. Representative confocal sections are shown. (**B**) Quantification of fluorescence intensity of acetylated H4K16 in control and siSirt2 oocytes. (n=10 for each group). (**C**) Young, old and old+Mel oocytes were immunolabeled with H4K16ac antibody (green) and counterstained with propidium iodide (red) for DNA. Representative confocal sections are shown. (**D**) Quantification of fluorescence intensity of acetylated H4K16 in young, old and old+Mel oocytes (n=10 for each group). Data are expressed as the mean ± SD from three independent experiments. *P<0.05 vs. controls. Scale bars: 5 μm.

SIRT2 has also been reported to be able to deacetylate histone H4 at lysine 16 (H4K16) to modulate chromatin condensation during cell cycle in mitosis [18, 30, 31]. Moreover, acetylation of H4K16 modulates nucleosome–nucleosome interactions and directly affects the correct chromosome segregation [32]. In specific, hypoacetylated H4K16 is important for maintaining the integrity of the kinetochore in mitotic cells [33]. Based on these findings, we proposed that SIRT2 controlled-H4K16 deacetylation pathway is likely involved in the effects of melatonin on oocyte quality. As shown in Figure 4A–4B, SIRT2 knockdown leads to the hyperacetylation of H4K16, consistent with the previous data [28]. Next we examined the state of H4K16 acetylation in young, old, old+Mel oocytes by immunostaining analysis. In support of our hypothesis, the acetylation level of H4K16 was significantly higher in aged oocytes than that in young cells. By contrast, melatonin supplement dramatically decreased the acetylation state of H4K16 in oocytes from reproductive aged mice (Figure 4C–4D). These results suggested that SIRT2-H4K16ac pathway is associated with the quality control of aged oocytes by melatonin.

### Acetylation status of H4K16 is essential for maintaining the meiotic structure in oocytes

In order to determine the function of H4K16 in oocyte meiosis, we generated plasmid in which lysine (K) 16 on histone H4 was mutated to arginine ® or glutamine (Q), and shall refer to the plasmids as H4K16R and H4K16Q mutants, respectively. Each of these amino acid substitutions changes the chemical character of lysine residue 16 on H4 in a different way. In the H4K16R protein, the long aliphatic side chain and the positively charged head that are feature of lysine are reserved, but the arginine cannot be acetylated. Whereas in the H4K16Q protein, the long aliphatic side chain with a polar head group of glutamine has chemical properties similar to an acetylated lysine and can therefore be regarded as a constitutive acetyl-mimic substitution [32]. It has been demonstrated that histone hyperacetylation can interfere with kinetochore assembly in somatic cells. Kinetochores are multiprotein complexes built on the centromeres of chromosomes [34]. Their ability to hold on to the ends of microtubules is crucial for the segregation of chromosomes to the daughter cells during cell division [35].

**Figure 5:**
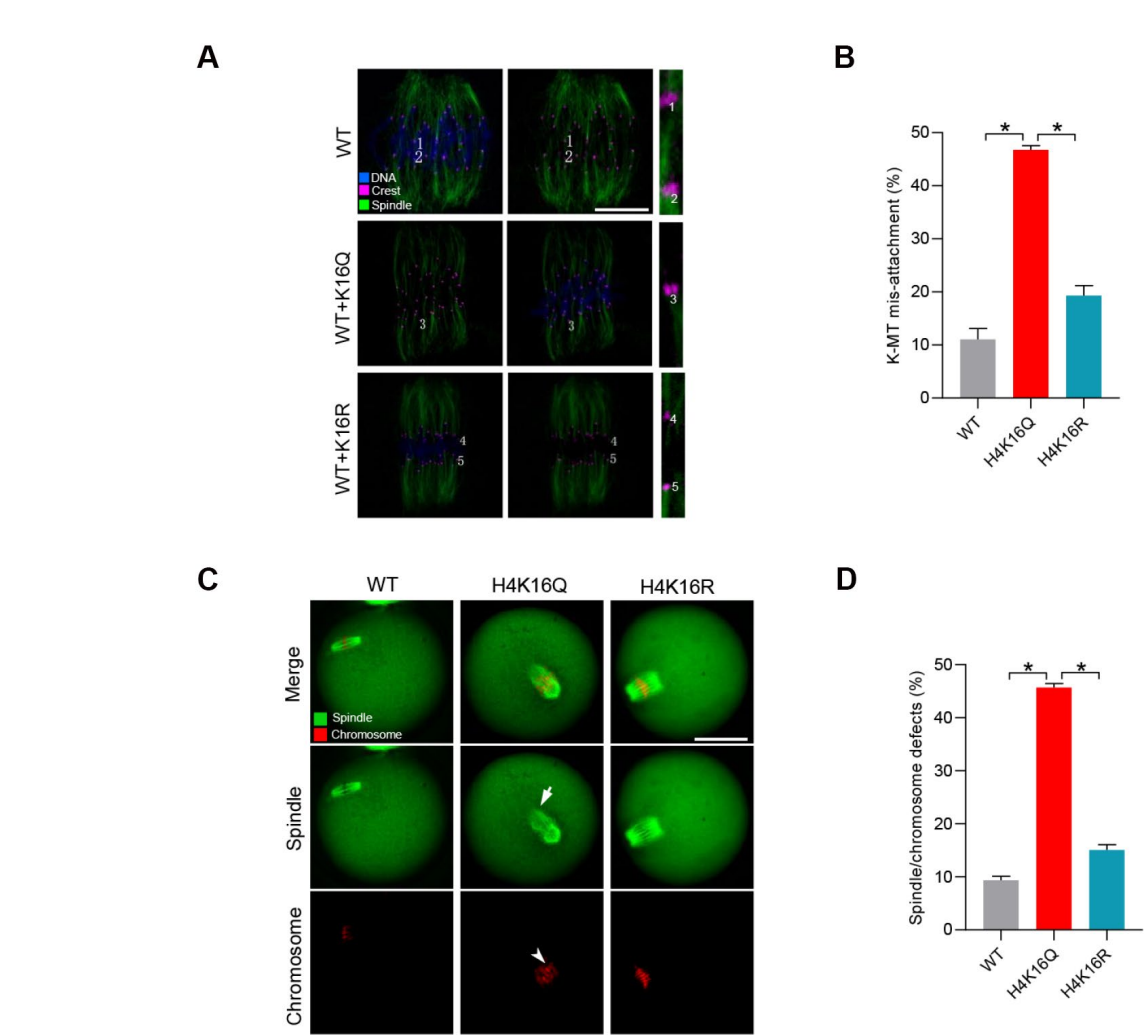
**Effects of H4K16 acetylation on kinetochore–microtubule attachment and meiotic apparatus in mouse oocytes.** (**A**) H4K16 wild-type (WT) and mutant-injected oocytes at metaphase stage were labeled with α-tubulin antibody to visualize spindle (green), CREST to detect kinetochore (purple), and co-stained with Hoechst 33342 for chromosomes (blue). Representative confocal sections are shown. Scale bars: 5 μm. Lost attachments were frequently detected in oocytes injected with H4K16Q mutant. (**B**) Quantitative analysis of K-MT mis-attachments in WT and H4K16 mutant-injected oocytes. Data are expressed as mean percentage ± SD from three independent experiments in which approximately 30 oocytes were analyzed. (**C**) WT and H4K16 mutant-injected oocytes were stained with α-tubulin antibody to visualize spindle (green) and counterstained with propidium iodide to visualize chromosomes (red). Representative confocal sections are shown. Scale bars: 50 μm. (**D**) Quantification of WT and H4K16 mutant-injected oocytes with spindle/chromosome defects. Data are expressed as mean percentage ± SD from three independent experiments in which around 115 oocytes were analyzed. *P<0.05 vs. controls.

Hence, we first evaluated the kinetochore-microtubule (K-MT) attachments in oocytes injected with H4K16Q and H4K16R mutant. It is interesting to note that the frequency of K-MT mis-attachment was readily detected in oocytes injected with H4K16Q (Figure 5A–5B). In contrast, H4K16R mutant has little effect on K-MT interaction during mouse oocyte meiosis. The persistence of K-MT attachment errors is the leading cause of chromosome mis-segregation. In line with this notion, we found the elevated incidence of spindle/chromosome disorganization in oocytes overexpressing H4K16Q as compared to H4K16R group (Figure 5C–5D). These observations suggest that H4K16 acetylation state is essential for the meiotic structure in oocytes by modulating the K-MT interaction.

### Melatonin alleviates maternal age-associated meiotic defects in oocytes through a SIRT2-dependent H4K16 deacetylation mechanism

**Figure 6:**
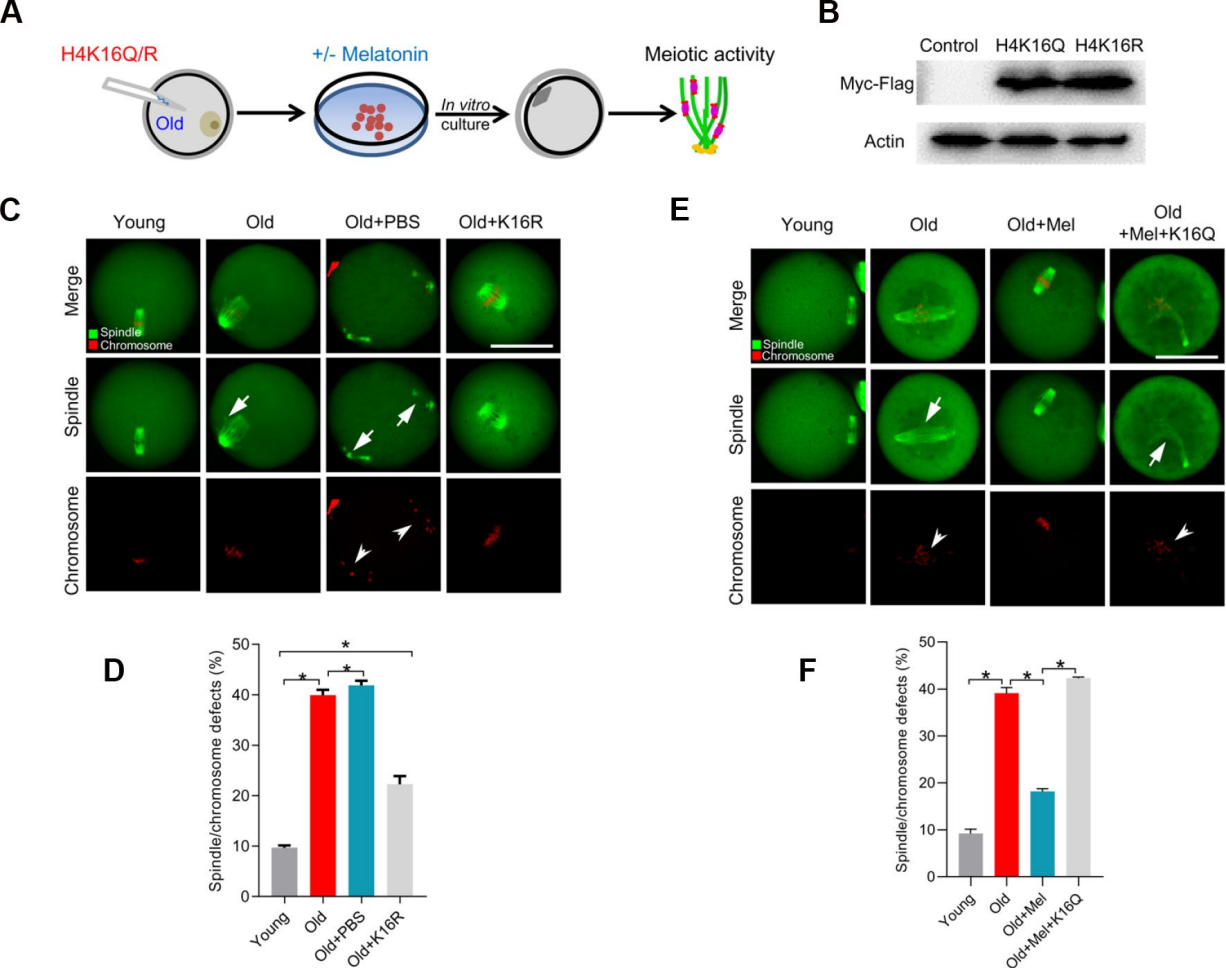
**Overexpression of H4K16R mutant ameliorates the maternal age-associated meiotic defects in mouse oocytes.** (**A**) Schematic illustration of the experimental protocol to check whether H4K16 acetylation mediates the effects of melatonin on the quality of aged oocyte. (**B**) Western blotting showing that two mutant H4K16 proteins were expressed to the similar extent. (**C**) Young, old, old+PBS and old+H4K16R oocytes were stained with α-tubulin to visualize spindle (green) and counterstained with propidium iodide to visualize chromosomes (red). Representative confocal sections are shown. Arrowheads indicate the misaligned chromosomes and arrows indicate the abnormal spindles. (**D**) Quantification of young, old, old+PBS and old+H4K16R oocytes with spindle/chromosome defects. (**E**) Young, old, old+Mel and old+Mel+H4K16Q oocytes were stained with α-tubulin to visualize spindle (green) and counterstained with propidium iodide to visualize chromosomes (red). Representative confocal sections are shown. Arrowheads indicate the misaligned chromosomes and arrows indicate the abnormal spindles. (**F**) Quantification of young, old, old+Mel and old+Mel+H4K16Q oocytes with spindle/chromosome defects. Data are expressed as mean percentage ± SD from three independent experiments in which at least 100 oocytes were analyzed. *P<0.05 vs. controls. Scale bars: 50 μm.

Given that melatonin could promote SIRT2 expression and H4K16 deacetylation in old oocytes, we hypothesize that SIRT2-H4K16ac pathway mediates the effects of melatonin on oocyte quality from reproductive aged mice. For this purpose, we first checked whether artificially altering the acetylation level at H4K16 would influence the beneficial effects of melatonin on aged oocytes (Figure 6A). Western blotting verified that exogenous H4K16 mutant was efficiently overexpressed in mouse oocytes, and the different mutants were expressed to the similar extent (Figure 6B). As we proposed, deacetylation-mimetic mutant H4K16R partially rescues the defective phenotypes of oocytes from reproductive aged mice (Figure 6C, 6D). On the other hand, following *in vitro* maturation, melatonin had no significant effects on the meiotic phenotypes in old oocytes overexpressing the acetylation-mimetic mutant H4K16Q (Figure 6E–6F). Taking together, these findings strongly suggest that melatonin alleviates advanced maternal age-associated meiotic defects in oocytes via a SIRT2–dependent H4K16 deacetylation pathway.

## DISCUSSION

In this study, we have uncovered a potential molecular mechanism showing how melatonin improves the quality of aged oocytes, specifically the meiotic phenotypes. The meiotic divisions in oocytes are highly error prone [36, 37]. Oocytes with the wrong number of chromosomes give rise to aneuploid embryos when fertilized. Chromosomal and spindle abnormalities become much more prevalent in oocytes with age and are considered the major factors responsible for the increased incidence of infertility, fetal loss and birth defects [24]. Herein, the high frequency of spindle defects and chromosome misalignment was observed in aged oocytes with reduced SIRT2 proteins (Figure 2). Therefore, loss of SIRT2 may be an important factor contributing to the meiotic abnormalities in oocytes from old mice. Melatonin content diminishes with age in serum, contributing to the aging-related pathologies [38]. Of note, we further found that melatonin supplementation either *in vivo* or *in vitro* could partly prevent the spindle/chromosome disorganization in matured oocytes from reproductive aged mice by increasing the expression of SIRT2 protein (Figures 1 and 2). Similarly, treatment of old rats with melatonin has been reported to not only ameliorate the oxidative stress but also concomitantly elevated SIRT2 expression in somatic cells [39]. Besides, melatonin also preserves the longevity SIRT1 expression in the hippocampus of total sleep-deprived rats [40]. Of note, melatonin is synthesized by mitochondria, especially in the oocytes, and it is a mitochondrial targeted molecule [41]. Therefore, these findings implicate that melatonin has a stimulatory effects on the expression of Sirtuin family protein, although the underlying mechanism remains to be clarified.

**Figure 7:**
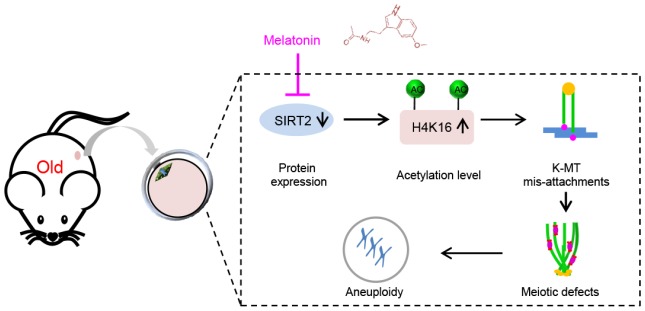
**Diagram illustrating the proposed mechanisms mediating the beneficial effects of melatonin on meiotic phenotypes of aged oocytes.** Melatonin supplement induces SIRT2 expression in oocytes from old mice, which in turn decreases the acetylation levels of H4K16, and thereupon promoting the establishment of proper kinetochore-microtubule attachment and the assembly of meiotic apparatus.

Emerging evidence suggests that melatonin is capable of improving quality of aged oocytes, mostly through the clearance of reactive oxygen species (ROS) and thereby maintaining redox homeostasis. In the present study, we reveal a novel molecular mechanism mediating the beneficial effects of melatonin on the competence of old oocytes: SIRT2 dependent-histone H4K16 deacetylation pathway. Multiple substrates of SIRT2 have been identified in various tissues and cell types. For example, histone H4K16 and α-tubulin-K40 have been reported to be the potential substrates of SIRT2, participating in the regulation of chromatin conformation and microtubule stability [18, 42]. Here we found that depletion of SIRT2 in mouse oocytes results in the hyperacetylation of H4K16 (Figure 4). H4K16 acetylation influences the folding of the chromatin fiber, and therefore SIRT2 deacettylase activity promotes the organization of chromatin conformation [43, 44]. It is worth noting that histone hyperacetylation can disrupt kinetochore assembly by destroying pericentromeric heterochromatin in somatic cells [45]. Likewise, hypoacetylation of H4K16 is critical for maintaining kinetochore function in budding yeast [39, 46]. Accurate chromosome alignment and separation depends on the correct attachment of kinetochores to microtubules emanating from opposite spindle pole [47]. Consistent with these observations, we demonstrated that overexpression of acetylation-mimetic mutant H4K16Q significantly increases the frequency of K-MT mis-attachments and spindle/chromosome defects during oocyte meiosis (Figure 5). Importantly, deacetylation-mimetic mutant H4K16R partially rescues the defective phenotypes of oocytes from reproductive aged mice (Figure 6). In addition, this study does not exclude that other substrates might be regulated by SIRT2 to influence meiotic apparatus in mouse oocytes. Recently, we found that melatonin supplement to the maturation medium directly induced the expression of SIRT3 protein in oocytes from obese mice [48]. The potential interaction between SIRT2 and SIRT3 in oocytes exposed to aging and obese environment needs further clarification. Altogether, our data reveal SIRT2-H4K16ac as an important mechanism affecting the meiotic structure of aged oocytes, and uncover the beneficial effects of melatonin on oocyte quality from reproductive aged mice by modulating this molecular pathway (Figure 7). More studies are needed to evaluate the therapeutic utility of melatonin for fertility issues associated with advanced maternal age.

## MATERIALS AND METHODS

### Animals and housing

Female ICR mice were used in this study. Mouse husbandry and all mouse experiments were carried out under the ethical guidelines of Nanjing Medical University (protocol #: IACUC-1601277). Mice were housed in ventilated cages on a 12-h light:12-h dark cycle at constant temperature (22 °C) and under specific pathogen-free conditions. To generate a natural aging mouse model, 42- to 45-week-old female mice which near the end of their reproductive lifespan were used. Mice were euthanized by cervical dislocation.

### Antibodies

Antibodies were purchased from the following sources: mouse monoclonal anti-α-tubulin-FITC antibody was purchased from Sigma (St. Louis, MO, USA; Cat#: T6074); rabbit polyclonal anti-SIRT2 antibody was purchased from Sigma (St. Louis, MO, USA; Cat#: S8447); mouse monoclonal anti-β-actin antibodies (St. Louis, MO, USA; Cat#: A5441) were purchased from Sigma; rabbit monoclonal anti-H4K16ac antibodies were purchased from Abcam (Cambridge, MA, USA; Cat#: ab109463); mouse monoclonal anti-Myc antibodies were purchased from Abcam (Cambridge, MA, USA; Cat#: ab18185).

### Melatonin treatment

For *in vivo* drug delivery, 42- to 45-week-old mice received melatonin (Sigma, Cat#: M5250) of daily oral doses of 30 mg/kg body weight in a corn oil carrier at 4:00 pm for 21 days before oocyte collection. The dose of melatonin was selected based on the published report [49] and our preliminary screening. For *in vitro* supplement, fully-grown oocytes from old mice were cultured in M16 medium containing 1 μM of melatonin. This concentration was selected based on the published literatures [48, 50].

### Oocyte collection and culture

Fully-grown GV oocytes were collected by removing cumulus cells in a drop of M2 medium through pipetting a few times with a glass capillary tube. For *in vitro* maturation, GV oocytes were cultured in M16 medium under mineral oil at 37°C in a 5% CO_2_ incubator. Ovulated oocytes were obtained by injecting mice intraperitoneally with 5 IU of Pregnant Mares Serum Gonadotropin (PMSG), and 48h later, with 5 IU of human chorionic gonadotrophin (hCG). Mice were euthanized 14h after hCG injection, and oocyte-cumulus complexes were collected from the oviducts and released into a hyaluronidase/M2 solution for the removal of cumulus cells.

### Short interfering RNAs

Short interfering RNAs (siRNAs) were purchased from Gene Pharma. For knockdown of SIRT2, a mix of the following siRNAs was used: sense 5′-GCAGCUUGUGUGAGCUCAATT-3′, antisense 5′- UUGAGCUCACACAAGCUGCTT-3′; negative control was used as a control.

### cRNA synthesis

The pCS2+ vector encoding the H4K16Q/R was generated with Quick Change site-directed mutagenesis kit (Strata gene) as previously described [51]. cRNA was transcribed *in vitro* from purified linear dsDNA templates. mMessage SP6 RNA polymerase kits (Invitrogen, Themo Fisher Scientific, USA) were used for the *in vitro* transcription reaction [52]. The primer sequences are listed in Supplementary Table 1.

### Microinjection

cRNA (10 ng/μL) purified on RNAeasy columns (Qiagen) was microinjected into GV oocytes with Eppendorf micromanipulators on a Nikon Eclipse Ti microscope and a FemtoJet microinjector. siRNAs were injected at a concentration of 1 mM. The injected volume was estimated to be 2 pL (estimated at 1% of the ooplasmic volume). After microinjection, oocytes were cultured in M16 medium supplemented with 2.5 μM milrinone for 20h under mineral oil at 37 °C in a humidified atmosphere of 5% CO_2_ in air.

### Protein extraction and Western blotting

Oocyte (100 per sample) lysates were extracted in Laemmli sample buffer with protease inhibitor and boiled for 5 minutes. The oocyte lysates were separated by SDS-PAGE in 12% gels. After electrophoresis, the separated proteins were transferred onto PVDF membrane. The membrane was blocked by incubation with 5% skim milk diluted by PBST for 1h at room temperature, and then incubated with primary antibodies overnight at 4°C. After washes in PBST, appropriate horseradish peroxidase-coupled secondary antibodies were used for chemiluminescence. The membrane was then washed in stripping buffer and reblotted with anti-actin (1:5,000) for loading control.

### Immunofluorescence

Oocytes were fixed with 4% paraformaldehyde for at least 30 min, followed by permeabilization with 0.5% Triton X-100 at room temperature for 20 min. After being blocked in 1% BSA-supplemented PBS for 1h, oocytes were immunostained using anti-α-tubulin, anti-SIRT2 or anti-H4K16ac antibody. Following an extensive wash with PBST, samples were incubated with secondary antibodies for 1h at room temperature. Chromosomes were evaluated by staining with propidium iodide (PI, 10 μg/ml) or Hoechst 33342 (5 μg/ml) for 10 min. Oocytes were mounted on anti-fade medium (Vectashield, Burlingame, CA, USA) and examined under a laser scanning confocal microscope (LSM 710, Zeiss, Germany). Fluorescent images were processed with ZEN software.

### Chromosome spread

Chromosome spread was carried out according to Wang et al [53] with minor modifications. In brief, zona pellucida of MII oocytes was removed by treatment with Tyrode’s buffer (pH 2.5) for 30s at 37°C. Then oocytes were individually plated onto glass slides pre-dipped in the fixative containing 1% paraformaldehyde and 0.15% Triton X-100. After air drying, oocytes were incubated with CREST overnight at 4°C and chromosomes were stained with Hoechst 33342. The laser scanning confocal microscope was used to examine chromosome numbers.

### Statistical analysis

Experiments were repeated at least three times. Statistical analyses were performed using Student’s *t* test and ANOVA as the circumstances may require. Differences with a P <0.05 were considered statistically significant.

## Supplementary Material

Supplementary Table 1
