# Supplementary material for: Melatonin ameliorates the advanced maternal age-associated meiotic defects in oocytes through the SIRT2-dependent H4K16 deacetylation pathway
Source: Aging (Albany NY). 2020 Jan 24;12(2):1610–23. doi: 10.18632/aging.102703 (PMC7053624; doi:10.18632/aging.102703)
Supplement: Supplementary Table 1 [file aging-12-102703-s001..pdf]

SUPPLEMENTARY TABLE

Supplementary Table 1. Primer sequences for cloning and site-specific mutation.

Gene	Primer sequence
H4	F: 5’ –ATGTCGGGTCGCGGCAAG–3’
	R: 5’ –GCCGCCGAATCCGTAGAG–3
H4K16Q	F: 5’ –GGCAAAGGCGGCGCTCAGCGCCACCGTAA–3’
	R: 5’ –GAGCGCCGCCTTTGCCCAGGCCTTTT–3’
H4K16R	F:5’ –GCAAAGGCGGCGCTAGGCGCCACCGTAA–3’
	R: 5’ –CTAGCGCCGCCTTTGCCCAGGCCTT–3’
